# Supplementary material for: Identification of photoperiod-induced specific miRNAs in the adrenal glands of Sunite sheep (Ovis aries)
Source: Front Vet Sci. 2022 Jul 22;9:888207. doi: 10.3389/fvets.2022.888207 (PMC9354845; doi:10.3389/fvets.2022.888207)
Supplement: Supplementary file 1 [file Data_Sheet_1.ZIP › Supplementary file/miRDeep2 file.zip!/miRDeep2 file/Novel_Structure/LP42A2/Novel_378.pdf]

Provisional ID	:	NC_019466.2_17600
Score total	:	3.2
Score for star read(s)	:	3.9
Score for read counts	:	-2.8
Score for mfe	:	0.6
Score for randfold	:	1.6
Score for cons. seed	:	
Total read count	:	6
Mature read count	:	4
Loop read count	:	0
Star read count	:	2

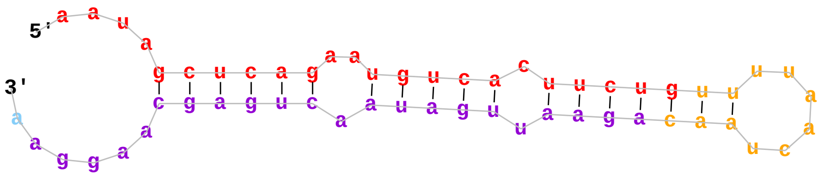

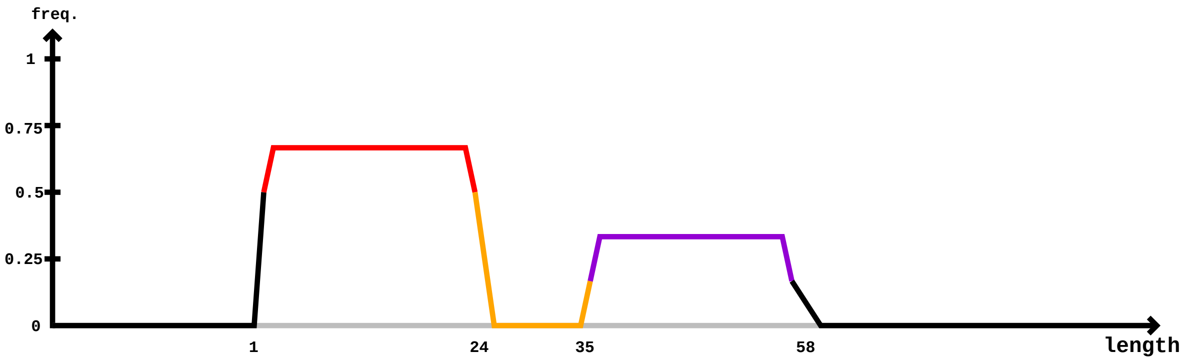

Mature **Star**

```
5'- uuuguaauucuuuacuguggaauagcucagaaugucacuucuguuuuaacuaacagaauugauaacugagcaaggaaacguaauuuggauuauaaaauucuugcuuuaauaaa   -3'    obs
    uuuguaauucuuuacuguggaauagcucagaaugucacuucuguuuuaacuaacagaauugauaacugagcaaggaaacguaauuuggauuauaaaauucuugcuuuaauaaa          exp
    ((((((((((.((((.((......((((((..(((((.(((((((......))))))).))))).))))))......))))))...)))))))))).................   reads      mm         sample
    ....................aauagcucagaaugucacuucug......................................................................   3          0          seq
    .....................auagcucagaaugucacuucu.......................................................................   1          0          seq
    ......................................................agaauugauaacugagcaagga.....................................   1          0          seq
    .......................................................gaauugauaacugagcaagg......................................   1          0          seq
```
